# Differences in the Microvascular Arrangement Lead to Improved Clinical Diagnostics of Esophageal Neoplasms: A Single-Center Retrospective Study

**DOI:** 10.3390/diagnostics14242852

**Published:** 2024-12-18

**Authors:** Ryogo Minami, Eriko Noma, Yoshiaki Moriguchi, Shinichiro Horiguchi, Toshiro Iizuka

**Affiliations:** 1 Department of Gastroenterology, Tokyo Metropolitan Cancer and Infectious Diseases Center, Komagome Hospital, 3-18-22 Honkomagome, Bunkyo-Ku, Tokyo 113-8677, Japan; minami373323@gmail.com (R.M.);; 2 Department of Pathology, Tokyo Metropolitan Cancer and Infectious Diseases Center, Komagome Hospital, 3-18-22 Honkomagome, Bunkyo-Ku, Tokyo 113-8677, Japan

**Keywords:** squamous intraepithelial neoplasia, squamous cell carcinoma, endoscopic diagnosis

## Abstract

Background/Objectives: Superficial esophageal cancer is diagnosed by evaluating the vascular architecture, including dilation, tortuosity, caliber change, and shape, of a lesion. However, this diagnosis is subjective and requires extensive experience. Endoscopically distinguishing squamous intraepithelial neoplasia (SIN) from esophageal cancer is difficult. Thus far, only a few studies have described the endoscopic findings of SIN. Therefore, the present study aimed to investigate whether endoscopic observation of the vascular architecture of tumors is useful in differentiating SIN from superficial esophageal cancer (SCC). Methods: This study included 141 patients who were histopathologically diagnosed with SIN or SCC between 2007 and 2023. Based on endoscopic images, patients were divided into those with a regular vascular arrangement (regular group) and those with an irregular vascular arrangement (irregular group). After evaluating the clinical characteristics, propensity score matching was used to assess the association between the groups and their pathological diagnoses. Results: Of the 141 patients, 44 and 97 were in the regular and irregular groups, respectively, with a ratio of 1:2. After propensity score matching, 33 and 66 patients were included in the regular and irregular groups, respectively. There were no significant differences between the groups after matching for age, alcohol consumption, smoking status, lesion site, sex, or lesion size. The regular group had significantly more patients with SIN, whereas the irregular group had significantly more patients with esophageal cancer (*p* < 0.001). Conclusions: The regularity of the vascular architecture may be useful for endoscopically distinguishing between SIN and esophageal cancer.

## 1. Introduction

In Japan, superficial esophageal cancer is routinely diagnosed endoscopically by evaluating the vascular architecture according to the Japanese Esophageal Association classification [1]. Esophageal cancer is diagnosed if well-demarcated changes consisting of dilatation, tortuosity, caliber change, and a distinctive shape (four signs) of vessels (type B1 vessels) are observed [1]. However, there are no commonly accepted criteria for endoscopically distinguishing squamous intraepithelial neoplasia (SIN), a neoplastic lesion of the esophagus that is not atypical enough to be considered cancerous, from superficial esophageal cancer. SIN is frequently encountered during regular upper endoscopic follow-up examinations for the early detection of multiple metachronous lesions in patients receiving treatment for esophageal cancer. However, thus far, only a few studies have described the endoscopic findings of SIN [2]. Moreover, empirically diagnosing SIN on the basis of endoscopic findings using the Japanese Esophageal Association classification, which relies solely on the appearance of the superficial vascular architecture, is difficult because such an evaluation is necessarily subjective and relies heavily on experience, making the assessment of the vascular architecture in borderline lesions very difficult.

Generally, patients are treated when the lesions progress from SIN to esophageal cancer [3]. Occasionally, a mixture of SIN and carcinoma is observed, which also complicates endoscopy-based diagnoses. Nonetheless, differentiating between SIN and esophageal cancer using endoscopic findings is important because it enables unnecessary biopsies and endoscopic procedures, with potential complications of perforation and bleeding.

When assessing the vascular architecture of a lesion to differentiate superficial esophageal cancer from SIN, a straight line connecting adjacent vessels is considered to indicate a regular arrangement of the vessels. The regularity of vessel arrangement is a more objective factor than dilatation, tortuosity, caliber change, or shape, which are used to describe type B1 vessels. Therefore, the present study aimed to determine whether the vascular arrangement is a useful endoscopic indicator of the differences between SIN and superficial esophageal cancer.

## 2. Materials and Methods

### 2.1. Study Design and Ethics Statements

This retrospective observational comparative case/control study was conducted in the Department of Gastroenterology at Tokyo Metropolitan Komagome Hospital and was approved by the institutional review board. Patient consent was waived because of the retrospective design of the study protocol.

### 2.2. Patients

**Figure 1:**
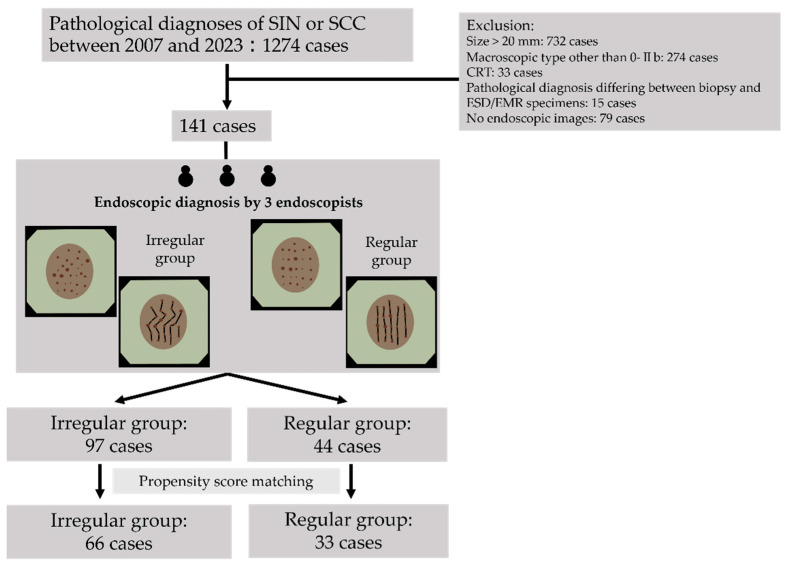
Overview of patient selection. SIN, squamous intraepithelial neoplasia; SCC, squamous cell carcinoma; CRT, chemoradiation.

Of the 1274 patients diagnosed with SIN or squamous cell carcinoma (SCC), based on the findings of an esophageal biopsy or endoscopic mucosal resection (EMR)/endoscopic submucosal dissection (ESD) specimens, between 2007 and 2023 at the study center, those who underwent narrow-band imaging (NBI) with magnifying endoscopy before biopsy or endoscopic treatment were included. The exclusion criteria were as follows: (1) a history of radiation to the pharynx or esophagus, (2) macroscopic type other than 0-IIb (all SIN lesions were 0-IIb on endoscopic diagnosis; patients with cancers other than 0-IIb were excluded), (3) the absence of pretreatment NBI with magnifying endoscopy, (4) the absence of endoscopic images for evaluating the vascular architecture of the area where the biopsy was performed, and (5) patients in whom the pathological diagnosis based on a biopsy specimen differed from that based on an ESD/EMR specimen. After applying these criteria, 141 patients were included in the analysis (Figure 1).

### 2.3. Endoscopic Procedure

Endoscopy was performed using either midazolam (0.2–1.0 mg; Dormicum, Maruishi Pharmaceutical, Tokyo, Japan), pethidine hydrochloride (17.5–35 mg; Pethidine, Takeda Pharmaceutical, Osaka, Japan), or dexmedetomidine hydrochloride (0.6 μg/kg/h; Precedex, Hospira Japan, Co., Osaka, Japan). The procedure was conducted under conscious sedation, and the choice of the sedation method was determined by the endoscopist. A high-definition magnifying endoscope (GIF-H260Z, GIF-H290Z, or GIF-XZ1200; Olympus Corporation, Tokyo, Japan) was used for all procedures.

### 2.4. Biopsy Protocol and EMR/ESD Procedure

A biopsy was performed on brownish areas >5 mm that were clearly demarcated under NBI without magnification if the endoscopist judged that cancer could not be excluded endoscopically using NBI with magnification. Before EMR/ESD, lesions were evaluated under white light, NBI without magnification, and NBI with magnification. After determining the extent of the lesion using iodine spray, the circumference of the lesion was marked with a DualKnife^®^ (Olympus Corporation, Tokyo, Japan). After marking, a glycerol solution (Chugai Pharmaceutical Co., Tokyo, Japan) containing a small amount of indigo carmine was injected into the submucosa using a hypodermic needle (01862; Top Corporation, Tokyo, Japan). During EMR, the lesion was aspirated using a cap attached to the endoscope tip, and the aspirated lesion was resected in the endocut mode using a snare. A needle-type ESD knife (DualKnife; Olympus) was used to make a circumferential incision and perform the dissection. These endoscopic procedures were performed by expert endoscopists certified by the Japanese Society of Gastrointestinal Endoscopy, who have performed >1000 procedures using NBI, or by trainee endoscopists under their supervision.

### 2.5. Pathological Diagnosis

The excised or biopsy specimens were stained with hematoxylin and eosin for pathological diagnosis. In accordance with the Japanese classification of esophageal cancer [4], SIN is defined as an intraepithelial lesion showing structural and cytological abnormalities indicative of a neoplasm; however, it does not include carcinoma in situ. It includes only low-grade dysplasia. The histopathological type (SIN or SCC), depth, and size of the lesions were assessed. Esophageal cancer was diagnosed even when a partially cancerous area was detected. The tumor invasion depth was classified according to the Japanese classification of esophageal cancer [4].

### 2.6. Definition and Evaluation of Vascular Arrangement

**Figure 2:**
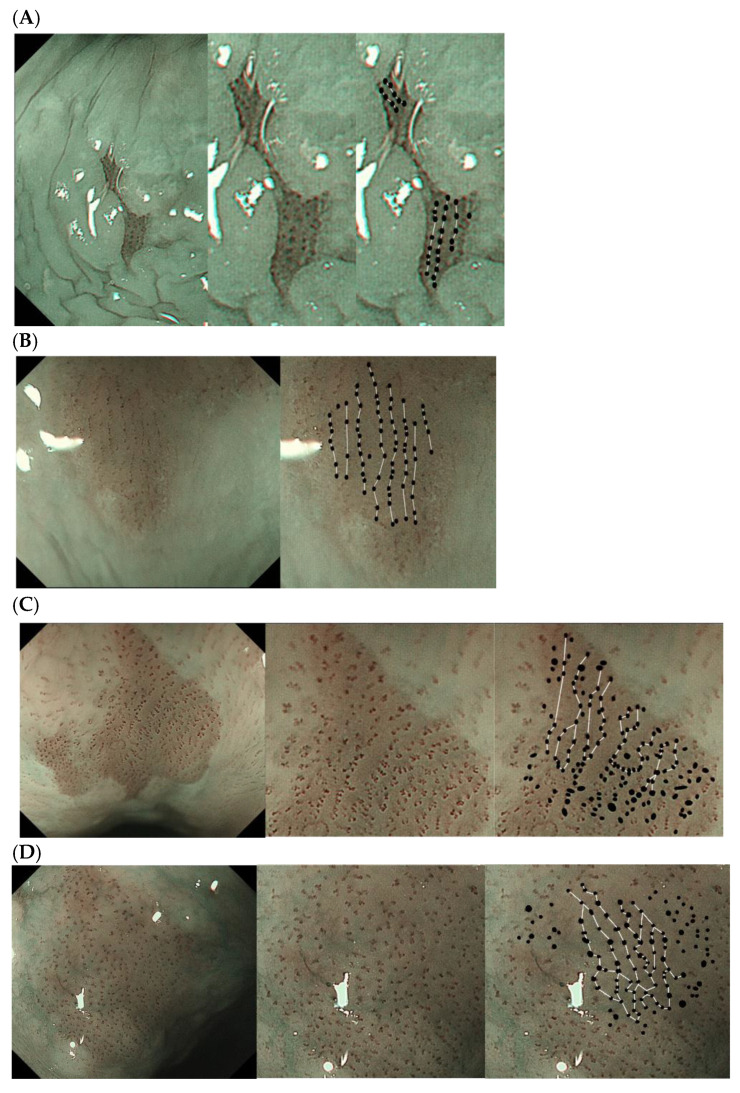
Endoscopic diagnoses. (**A**,**B**) Linear vascular arrangement observed on magnifying NBI endoscopy classified as regular. (**C**,**D**) Non-linear vascular arrangement with irregular intervals between vessels classified as irregular. NBI, narrow-band imaging.

The vascular arrangement of a tumor was considered “regular” if the vessels, regardless of type, occurred at regular intervals from each other and if NBI with magnification showed a linear, vascular pattern. In contrast, the vascular arrangement of a tumor was considered “irregular” if the intervals between the vessels were irregular and if NBI with magnification did not show a linear pattern of connections (Figure 2).

Three endoscopists (with ≥10 years of endoscopic experience) who were blinded to the clinicopathological information determined whether the vascular architecture of a lesion was regular or irregular on the basis of the endoscopic images of the lesion or the biopsied site. If a lesion had a heterogeneous vascular architecture comprising regular and irregular areas, it was considered irregular. In cases where the opinions of the assessors differed, the final assessment was determined by a majority vote.

### 2.7. Outcomes

The primary endpoint was whether the vascular architecture of a tumor (regular or irregular) was sufficient to endoscopically distinguish between esophageal cancer and SIN.

### 2.8. Statistical Analysis

Variables are expressed as the mean ± standard deviation, median, and interquartile range or percentage. Propensity score (PS) analysis was performed as a non-randomized sensitivity analysis to control for and reduce selection bias per group. The PS was estimated using multivariable logistic regression, with age, alcohol consumption (g/week), and smoking status (pack years) as covariates. In the Japanese population, esophageal cancer occurs most commonly in the middle-aged population [5], and alcohol consumption and smoking are known risk factors [6]. Therefore, age, alcohol consumption, and smoking status were selected as covariates. The match ratio was 1:2, and the nearest-neighbor matching method was used (caliper width, 0.2).

Then, the pathological diagnoses were compared. Differences in baseline characteristics between the regular and irregular groups were assessed using the Student’s *t*-test for continuous variables with a normal distribution, a chi-square test or Fisher’s exact test for categorical variables, and the Wilcoxon rank-sum test for rank variables and continuous variables with a non-normal distribution, as appropriate. A *p*-value < 0.05 was considered to indicate statistical significanceand all statistical analyses were performed using EZR; https://www.jichi.ac.jp/saitama-sct/SaitamaHP.files/statmed.html (accessed on 1 November 2024), Kanda 2012 [7]. More precisely, it is a modified version of the R commander designed to add statistical functions frequently used in biostatistics.

## 3. Results

### 3.1. Patient Characteristics

Three endoscopists blinded to the clinicopathological information divided the 141 patients into regular and irregular groups on the basis of the endoscopic findings of the vascular architecture. Forty-four and ninety-seven patients were included in the regular and irregular groups, respectively (Figure 1). Table 1 shows the clinical characteristics of the patients before and after PS matching. After PS matching, 33 and 66 patients were included in the regular and irregular groups, respectively. Background factors, especially age, alcohol consumption, smoking status, lesion site, and proton pump inhibitor use, were adjusted for to reduce bias. No significant differences in other baseline characteristics were observed between the groups.

### 3.2. Inter-Observer Agreement Regarding Vascular Arrangements

**Figure 3:**
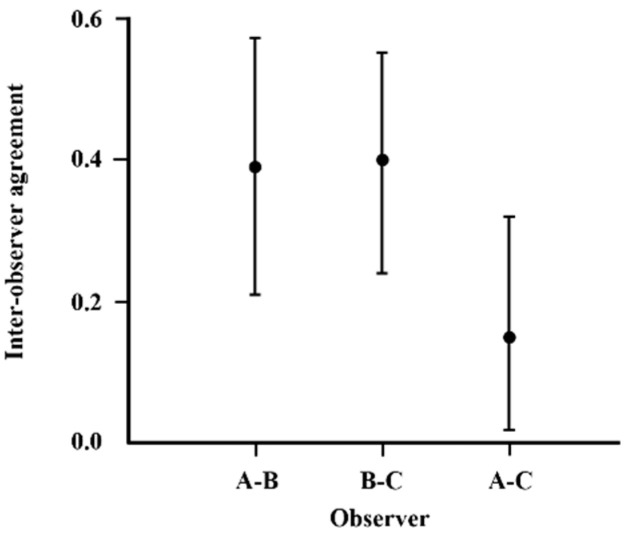
Inter-observer agreement for endoscopic diagnoses (regular or irregular vascular arrangement). Kappa values and 95% confidence interval are presented.

Three endoscopists performed blind evaluations to assess the degree of inter-observer agreement. In cases of disagreement, the measurement of the degree supported by the majority of evaluators was adopted. Figure 3 shows the degree of inter-observer reliability. The kappa coefficients for endoscopists A and B, A and C, and B and C were 0.39, 0.40, and 0.15, respectively (Figure 3).

### 3.3. Pathological Characteristics of Patients Included After PS Matching

After PS matching, 64 patients with esophageal cancer (SCC) and 35 patients pathologically diagnosed with SIN were included in this analysis. Their pathological characteristics are shown in Table 2. No significant difference was found between SCC and SIN in terms of the lesion site (*p* = 0.59). The lesion size of SIN was significantly smaller than that of SCC (12.8 mm versus 8.5 mm; *p* < 0.001) (Table 2).

### 3.4. Comparison Between Regular and Irregular Groups

Analysis of the degree of association between the endoscopic and pathological diagnoses of the tumors in the regular and irregular groups revealed that the former had significantly more cases of SIN, whereas the latter had significantly more cases of SCC (*p* < 0.001) (Table 3). The sensitivity of this endoscopic diagnosis in differentiating SIN from SCC was 0.60, with a specificity of 0.81.

## 4. Discussion

To the best of our knowledge, the present study is the first to show that the degree of regularity in the vascular architecture of tumors is useful for endoscopically differentiating SIN from SCC. When discussing cancer and precancerous lesions, it is important to specify the definition by which a diagnosis is made. Japanese and Western pathologists assume different positions regarding the histological diagnosis of cancer. Stromal invasion is considered the most important diagnostic criterion in the West, whereas the degree of atypia of the cell nuclei is considered more important in Japan [8,9,10,11,12]. In the diagnosis of precancerous lesions of esophageal cancer, the current World Health Organization (WHO) classification (5th edition) classifies intraepithelial neoplasia (IN) as low- or high-grade [13], whereas the current Japanese classification of esophageal cancer classifies most cases of high-grade IN as CIS [4]. Here, the presence of an atypical epithelium was insufficient as a criterion for cancer but formed the basis of the diagnosis of SIN, which corresponds to the classification of IN in the current Japanese classification of esophageal cancer, low-grade IN in the WHO classification (5th edition) [13], and category 3 in the revised Vienna classification [3] (Table 4). In these cases, follow-up observations are optional.

Endoscopically detected IN, corresponding to category 3 in the revised Vienna classification, can be followed up without immediate treatment. The diagnosis of superficial esophageal cancer, in accordance with the Japan Esophageal Society classification [1] using high-magnification endoscopy, is mainly based on the appearance of superficial and vascular architectural features, including dilatation, tortuosity, caliber change, and shape. If the vessels demonstrate all four of these features, esophageal cancer is diagnosed. If all four features are absent, either low-grade IN or inflammatory IN is diagnosed. Although some lesions have the characteristics of type B vessels, it is difficult to determine whether all four features are present. Moreover, a detailed evaluation of the vascular architecture is sometimes difficult to perform within the limited time available during a procedure, especially in the presence of salivary secretion, gastric acid reflux, and esophageal peristalsis. Using the vascular architecture (regular or irregular) as a basis for endoscopically determining whether a lesion is an IN or a revised Vienna classification category 3 or 4 lesion (for which treatment is indicated) is simpler and more practical. The present study found that focusing on the vascular architecture within a lesion rather than focusing on the individual vascular architecture as per the conventional method was more useful for endoscopically differentiating between SIN (category 3) and superficial esophageal cancer (category 4). The kappa values for inter-observer agreement were 0.15, 0.39, and 0.40, indicating moderate agreement to a degree that is acceptable in clinical practice.

Reports of endoscopic findings distinguishing superficial esophageal carcinoma and high-grade IN from low-grade IN have mostly focused on vascular architecture and intervascular background coloration [2,14,15]. Mochizuki et al. reported that brownish dots and an atypical epithelium were significantly less common in low-grade IN than in high-grade IN in esophageal lesions with a diameter of ≤10 mm on NBI [16]. The present study also found that IN was smaller than superficial esophageal cancer. Furthermore, low-grade IN (category 3) with dilated vessels and a brownish epithelium was occasionally encountered. However, there have been no studies on the regularity of the vascular arrangement in such cases. In normal esophageal mucosa, loop-like vessels arise from the subepithelial capillary network beneath the epithelium. These microvessels, which exist within the epithelial papillae and are intrapapillary capillary loops (IPCLs) [14], can be clearly visualized via magnifying endoscopy with NBI. Kubota et al. [17] reported that the vascular architecture characteristics of esophageal cancer (dilation, tortuosity, caliber change, and varied shape) are mainly due to the dilation and extension of the IPCLs. The authors stained tissue from normal esophagus, esophagitis, low-grade IN, and high-grade IN cases with a CD105 antibody, which selectively stains tumor vessels, and then measured the vessel density. They found that CD105-positive vessels emerged from esophagitis and that the density of the tumor vessels increased as atypia increased. Thus, they succeeded in endoscopically observing modifications in the existing IPCLs, as well as newly formed abnormal vessels. The newly formed capillaries demonstrate a looping configuration that resembles immature IPCL-like capillaries (IPCL-like abnormal capillaries) [18]. It is hypothesized that in IN, the induction of IPCL-like abnormal capillaries occurs only within the existing IPCL and that the vascular arrangement is regular until an increase in the density of the abnormal vessels distorts the vascular architecture. Although it is difficult to demonstrate a 1:1 match between endoscopically observed vessels and those found in histological specimens, the hypothesis may be considered a possibility.

The present study has several limitations. First, this was a monocentric retrospective study. Thus, these findings require verification in a multicenter prospective study to assess their utility in clinical practice. Verification at other facilities and in other countries may show different results. Second, the endoscopic images were selected by endoscopists, thereby introducing potential selection bias. Additionally, endoscopic diagnosis was performed by doctors at the same facility, which may introduce a bias. Third, the vascular architecture was not assessed during endoscopy; thus, the findings may differ from those of studies that used real-time endoscopic diagnoses. Finally, partial disruption of the vascular architecture was judged to indicate an irregular pattern because matching the endoscopic and pathological images of the vessels in all patients was difficult, and cancer was diagnosed if even a small portion of cancerous tissue was detected in a histological specimen composed predominantly of IN. It is possible that in some patients, the endoscopic findings did not strictly contrast with the pathological findings.

## 5. Conclusions

The regularity of the vascular architecture may be useful for endoscopic differentiation between superficial esophageal cancer and IN.

## Figures and Tables

**Table 1:** The clinical characteristics of the patients in this study before and after propensity score matching.

	Before Matching			After Matching		
	Regular (*n* = 44)	Irregular (*n* = 97)	*p*-Value	Regular (*n* = 33)	Irregular (*n* = 66)	*p*-Value
Age, mean ± SD	73.3 ± 9.87	71.3 ± 9.34	0.278	72.9 ± 9.00	72.6 ± 8.71	0.866
Sex, *n* (%)			0.812			0.789
Male	36 (81.8)	81 (83.5)		26(79)	54(82)	
Female	8 (18.2)	16 (16.5)		7 (21)	12(18)	
Lesion location, *n* (%)			0.082			0.246
Cervical esophagus	3 (6.8)	7 (7.2)		2 (6.1)	2 (3.0)	
Upper esophagus	5 (11.4)	29 (29.9)		4 (12.1)	19 (28.8)	
Middle esophagus	27 (61.4)	42 (43.4)		19 (57.6)	30 (45.5)	
Lower esophagus	9 (20.5)	19 (19.6)		8 (24.2)	15 (22.7)	
Lesion diameter (mm)	10.8 (3–20)	10.8 (2–20)	0.983	11.1 (3–20)	11.4 (2–20)	0.84
Smoking, pack years	28.4 (0–110)	27.8 (0–162)	0.911	26.4 (0–100)	25.9 (0–162)	0.923
Alcohol, g/week	293 (0–1000)	327(0–1400)	0.495	308 (0–630)	294(0–1400)	0.786
Alcohol, years	36 (0–70)	42 (0–74)	0.118	39 (0–70)	41 (0–74)	0.727
PPI use, *n* (%)	15 (34)	27 (28)	0.551	11 (33)	22 (33)	1.00
Prior iodine staining, *n* (%)	0 (0)	0 (0)	1.00	0 (0)	0 (0)	1.00
Past history: esophageal	23 (52)	50 (52)	1.00	20 (61)	33 (50)	0.394
EMR/ESD, *n* (%)						

PPI, proton pump inhibitor; EMR, endoscopic mucosal resection; ESD, endoscopic submucosal dissection; SD, standard deviation.

**Table 2:** Pathological characteristics after propensity score matching.

	Squamous Cell Carcinoma	Squamous Intraepithelial Neoplasia	*p*-Value
Number of lesions	64	35	
Lesion location			0.59
Cervical esophagus	2	2	
Upper esophagus	17	6	
Middle esophagus	32	17	
Lower esophagus	13	10	
Lesion diameter (mm), median (range)	12.8 (3–20)	8.5 (2–20)	<0.001
Macroscopic type: 0-IIb	64	35	
Depth of invasion, *n*			
EP	46	-	
LPM	16	-	
MM-SM1	2	-	
Tissue sampling, *n*			<0.001
EMR + ESD	64	20	
Biopsy	0	15	

EP, epithelium; LPM, lamina propria mucosae; MM-SM1, muscularis musosae-submucosa1 (within 200 μm of muscularis mucosae); EMR, endoscopic mucosal resection; ESD, endoscopic submucosal dissection.

**Table 3:** A comparison of the pathological diagnoses between the regular and irregular groups.

		Endoscopic Diagnosis		*p*-Value
		Regular	Irregular	
Pathological diagnosis	SIN	21	14	
	SCC	12	52	<0.001

SIN, squamous intraepithelial neoplasia; SCC, superficial esophageal cancer.

**Table 4:** Revised Vienna classification of gastrointestinal epithelial neoplasia.

Category	Diagnosis	Clinical Management
1	Negative for neoplasia	Optional follow-up
2	Indefinite for neoplasia	Follow-up
3	Mucosal low-grade neoplasia	Endoscopic resection or follow-up
	Low-grade adenoma	
	Low-grade dysplasia	
4	Mucosal high-grade neoplasia	Endoscopic or surgical local resection
	4.1 High-grade adenoma/dysplasia	
	4.2 Non-invasive carcinoma	
	4.3 Suspicious for invasive carcinoma	
	4.4 Intramucosal carcinoma	
5	Submucosal invasion by carcinoma	Surgical resection

## Data Availability

The de-identified data presented in this study are available upon request from the corresponding author as per institutional review board requirements.
